# Pelvic Lymph Node Lymphangiomyomatosis Found During Surgery for Gynecological Fallopian Tube Cancer: A Case Report and Literature Review

**DOI:** 10.3389/fmed.2022.917628

**Published:** 2022-07-15

**Authors:** Shan Xiao, Yijia Chen, Qianjue Tang, Lianwei Xu, Li Zhao, Zhenzhen Wang, Erkai Yu

**Affiliations:** Department of Gynecology, LongHua Hospital Affiliated to Shanghai University of Traditional Chinese Medicine, Shanghai, China

**Keywords:** lymphangiomyomatosis, gynecological surgery, fallopian tube carcinoma, pelvic lymph nodes, case report

## Abstract

**Background:**

Lymphangioleiomyomatosis (LAM) is a rare low-grade metastatic tumor with an unknown origin that spreads through lymphatic vessels. It is characterized by the proliferation of smooth muscle-like or epithelioid tumor cells in the lung and axial lymphatic system. Extrapulmonary LAM is a localized disease with a low incidence rate, and the location of the related lesions is atypical. It is difficult to diagnose. The LAM of pelvic lymph nodes is hidden. It is usually found through gynecological oncology surgery.

**Case presentation:**

We report a 57-year-old postmenopausal woman with a pelvic mass and vaginal bleeding as the main symptoms. The patient had no history of pulmonary LAM, tuberous sclerosis complex (TSC), or renal angiomyolipoma and had not used exogenous hormones. We performed a total hysterectomy, bilateral adnexectomy, greater omentum resection, and pelvic lymphadenectomy under laparoscopy. The postoperative pathology confirmed high-grade serous carcinoma of the left fallopian tube, and four lymph nodes were found in the pelvic lymph nodes, suggesting lymphangiomyomatosis. Immunohistochemical results also showed that these cells could express markers of smooth muscle cells and melanoma cells. The patient was treated with chemotherapy after the operation. Chest CT did not suggest lung LAM during the postoperative follow-up, and there was no tumor recurrence.

**Conclusion:**

The diagnosis of this disease is challenging. At the same time, due to insufficient clinical samples, it is still unknown whether there is a potential relationship between pelvic and peritoneal lymph node LAM found in the surgical staging of gynecological tumors and lung LAM and/or TSC. There is no evidence that pelvic and peritoneal lymph node LAM will increase the risk of pulmonary LAM. Therefore, additional clinical data are required to analyze and summarize the relationship between pelvic and peritoneal lymph node LAM, pulmonary LAM, and the source of LAM. We present a case of pelvic lymph node LAM and propose a hypothesis that the pathogenesis of endometriosis can be used for reference in the study of this disease.

## Introduction

Lymphangiomatosis, also known as lymphangioleiomyomatosis (LAM), is a tumor composed of lymphangiomyocytes proliferating around lymphatics, sometimes with lymphocyte aggregation. Focal lesions are called lymphangiomyomas, and those with or without the involvement of pulmonary parenchyma are called lymphangiomyomatosis. Von Stossel first reported it in 1937. It belongs to one of the lesion families collectively known as perivascular epithelial cell tumors (PEComa). It is a slow-moving, low-grade, metastatic systemic disease characterized by progressive cystic lung destruction, abdominal tumors, and chylous fluid accumulations, including angioleiomyoma and lymphangioleiomyoma (1, 2). This disease is rare and easily misdiagnosed. It occurs almost exclusively in women, mainly in those of childbearing age. According to statistics, approximately 3.4–7.8 people per million women suffer from LAM (3). LAM can be distributed or concurrent based on TSC. LAM is most common in the lung. LAM lesions found in pelvic and peritoneal lymph nodes are rare and hidden. At the same time, LAM lesions have been reported in the uterus (4). Whether lesions in the uterus are metastatic or the source of cancer has always been the focus of debates. Primary extrapulmonary LAM is extremely rare. According to the currently reported cases, pelvic lymph node LAM found in gynecological surgery is usually uterine body cancer, cervical cancer, ovarian cancer, hysteromyoma, endometriosis, adenomyosis, and other diseases. To the best of our knowledge, primary pelvic lymph node LAM found in primary fallopian tube cancer surgery is relatively rare. Here, we report a case of pelvic lymphangiomyomatosis in the staging operation of gynecological fallopian tube cancer, and there was no pulmonary LAM at the 12-month postoperative follow-up.

## Case Presentation

**Figure 1:**
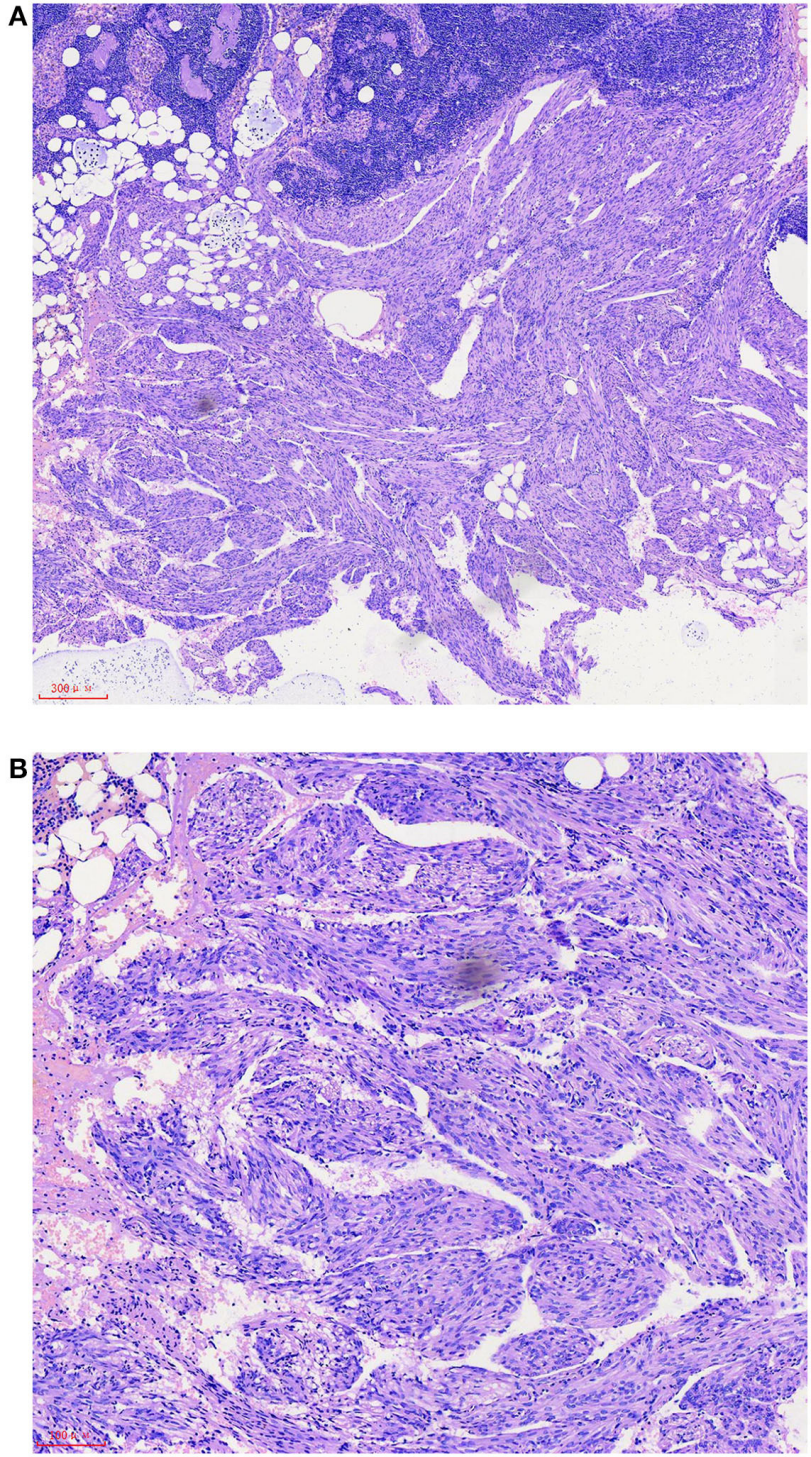
After hematoxylin-eosin (HE) staining, it is found that there is a fascicular proliferative smooth muscle-like spindle cell area outside the pelvic lymph nodes, which has a clear boundary with the surrounding tissues **(A)**. Smooth muscle-like spindle cell nuclei have no atypia and are mostly arranged around reticular or sinusoidal cavities lined with flat endothelium. Residual lymph nodes can be seen in local areas between proliferative spindle cells **(B)**. The scale represents 300 μm **(A)** and 100 μm **(B)**.

**Figure 2:**
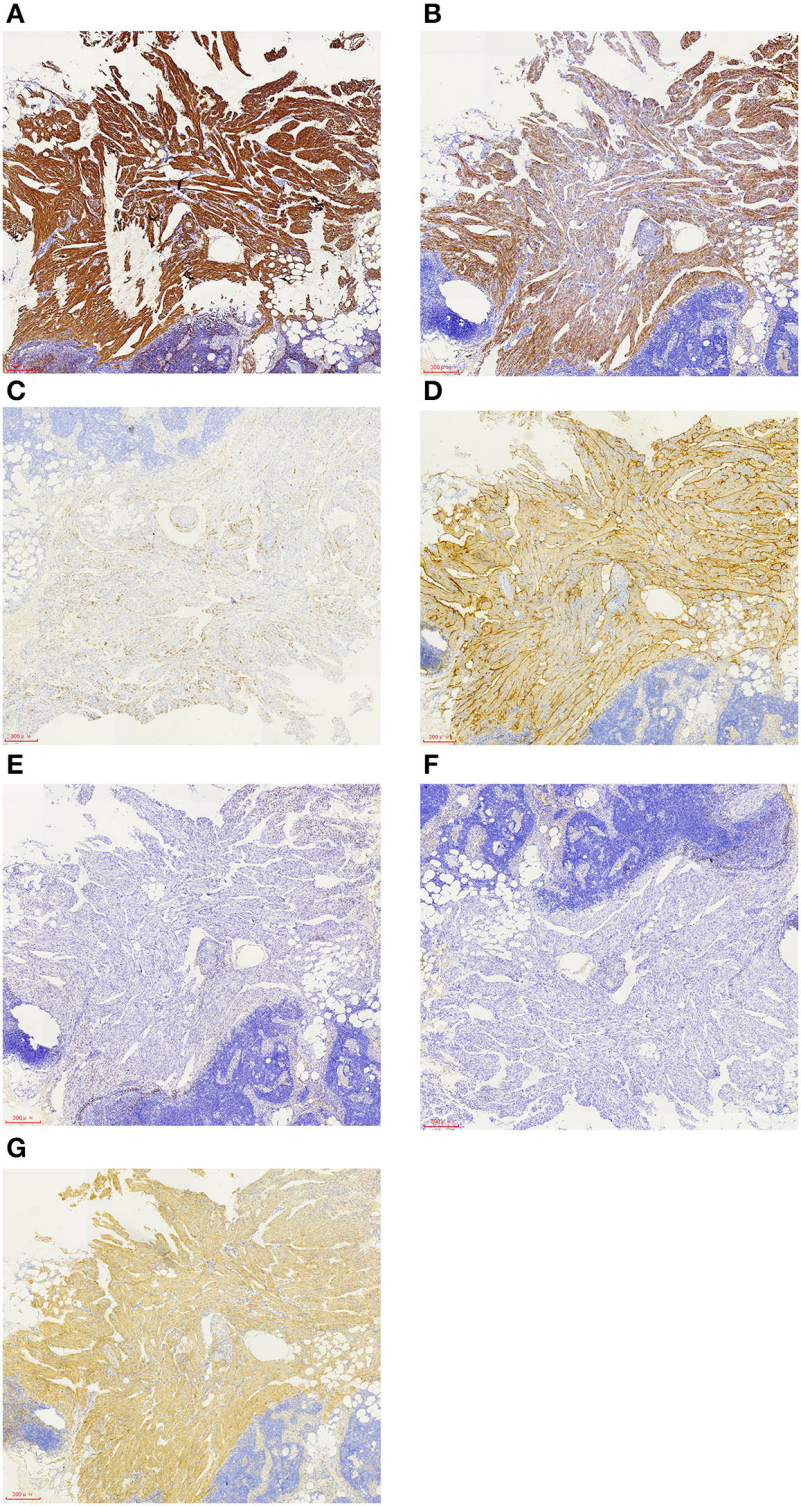
Immunohistochemistry of LAM cells showed that the smooth muscle-like specific protein markers a-SMA **(A)** and desmin **(B)** were positive, the melanocyte marker HMB45 was focally positive **(C)**, D2-40 was positive **(D)**, ER and PR were negative **(E,F)**, and β-catenin was positive **(G)**. The scale represents 300 μm **(A**–**G)**.

A 57-year-old female patient was hospitalized for “one year of menopause, pelvic mass with irregular vaginal bleeding for 3 weeks.” The patient had a history of well-controlled hypertension, denied a relevant family, and disease history. There is no history of lung disease, exogenous hormone use, TSC, or renal angiomyolipoma. 1-0-0-1, spontaneous labor. The patient was admitted to the hospital for an ultrasound examination, which showed that the intima was 7.3 mm, and the echo of the intima was uneven. There was a mixed echo on the left side of the pelvic cavity, with a size of 99 mm × 48 mm, which was mainly hypoechoic with a pipe-like structure. Preoperative PET-CT examination revealed an irregular cystic, solid mass on the left side of the pelvis, an abnormal increase in FDG metabolism in the solid part in front of the mass, the maximum value of SUV is 6.51, 7.02 after delay, the uptake range is 38 mm × 25 mm and possible large malignant lesions from the left appendix. Preoperative chest CT showed a small amount of pleural effusion on both sides, and no apparent abnormalities were found in the rest. The results of the preoperative pulmonary function examination were normal. We performed a laparoscopic total hysterectomy, bilateral adnexectomy, greater omentum resection, and pelvic lymphadenectomy. During the operation, the left fallopian tube was thickened and twisted, approximately 15 cm long, and brittle tumor tissue was seen at the fimbriae end. The uterus is slightly enlarged, and a small intramural myoma was seen in the posterior wall. There was no right fallopian tube abnormality and bilateral ovarian atrophy. The left pelvic wall adhered to the intestinal canal, and the greater omentum adhered to the abdominal wall. There was no evident lymphadenopathy. The postoperative pathology showed that the malignant epitheliogenic tumor of the left adnexa was consistent with high-grade serous carcinoma, and the possibility of fallopian tube origin was high. No definite vascular and nerve recidivism. No tumor metastasis was found in the bilateral parauterine and cervical margins. One of the eight lymph nodes in the left pelvic cavity showed lymphangiomyomatosis, 2 mm in diameter. Among the seven lymph nodes in the left obturator, two lymph nodes showed lymphangiomyomatosis, 5 mm in diameter. Among the five lymph nodes in the right obturator, one lymph node is seen, showing lymphangiomyomatosis, 5 mm in diameter. See in (Figures 1A,B) for the H&E staining results of the lymph node LAM pathological sections. Immunohistochemistry showed a-SMA positivity, Desmin positivity, HMB45 (scattered cells +), D2-40 (vessels +), β-catenin (cytoplasmic +), ER, and PR were negative (see Figures 2A–G).

According to the pathological report after the operation, the patient was diagnosed with high-grade serous carcinoma of the left fallopian tube (p-T1aN0M0 stage IA). We gave the patient a cycle of chemotherapy with paclitaxel and carboplatin. After chemotherapy, the patient had no discomfort or bone marrow suppression, so she was discharged from the hospital. The follow-up patient elected to stop treatment because of pronounced nausea and vomiting after chemotherapy. As of 7 April 2021, the patient was followed up in our hospital again. Nearly 8 months had passed since the operation and chemotherapy. A small amount of pleural effusion was still seen on chest CT, similar to before the operation. There were no apparent abnormalities in others, and there was no evident recurrence on upper abdominal enhanced CT and pelvic enhanced MRI. The patient was followed up for 12 months after the operation, and there were no related pulmonary symptoms.

## Discussion

Lymphangiomyomatosis (LAM) is a rare and slow-moving multisystem disease. The common manifestation is abnormal smooth muscle-like cells infiltrating the lung parenchyma, resulting in cystic destruction of the lung (1). LAM is most common in the lungs, and its extrapulmonary features are mainly angiomyolipoma and lymphatic abnormalities. Angiomyolipomas are benign mixed mesenchymal tumors most often found in the kidney. They can be found in approximately 30–40% of women with sporadic LAM and 88–96% of women with TSC-LAM (5, 6), and most of these tumors are small and asymptomatic (7). Lymphatic manifestations include the formation of lymphangioleiomyoma and chylous pleural and ascites, occasional chylous pericardial effusion, chylous hemoptysis, and chyluria (1).

The cell source of this disease is unknown and often complicated with TSC. TSC is a multisystem autosomal dominant disease caused by germline mutations of the TSC gene, TSC1, or TSC2. Gene mutation can lead to the constitutive activation of the rapamycin (mTOR) mechanical target pathway, the imbalance of LAM cell growth and proliferation, and the obstruction of lymphangiogenesis, resulting in a lung remodeling disorder (1). LAM cells have the appearance of “immature” smooth muscle-like cells. They can express smooth muscle-like specific proteins (α-smooth muscle actins, Desmin) and the melanoma cell marker gp100, in which gp100 can be recognized by the antibody HMB45 (human melanoma black 45) (8, 9). In this case, the immunohistochemical hints of LAM in the patient's lymph nodes α-SMA, Desmin, and HMB-45 were all positive (see Figures 2A–C), which was consistent with the characteristics of LAM cells.

In addition to the overactivation of the mTOR pathway, LAM may also have a metastasis mechanism. The study found that LAM recurrence occurred after single lung transplantation, indicating that the metastasis mechanism may be involved in the progression of LAM disease (10). Szpurek et al. reported a case of a 47-year-old female primary uterine LAM with ovarian and lung metastasis (11), indicating that although LAM is considered benign histologically, LAM has the biological tendency to transform into a malignant tumor. LAM cells have the ability to grow, migrate, and invade, enter the lungs through lymphatic circulation, and even destroy distant tissues (12). However, due to the long duration of the disease, patients can remain in good condition for several years.

LAM is difficult to diagnose. The diagnostic features include TSC, angiomyolipoma, chylous pleural effusions or ascites, and lymphangioleiomyomas (2). For patients whose chest CT shows LAM lesions but there is no other confirmed evidence, it is recommended to detect vascular endothelial growth factor D (VEGF-D) before lung biopsy (2). The guidelines (2) recommend that patients with LAM be treated with sirolimus, but the guidelines do not advocate hormone therapy, including progesterone, gonadotropin-releasing hormone agonist, selective estrogen receptor modulator, and oophorectomy, because most patients do not show beneficial results.

Extrapulmonary LAM is usually a benign lesion, and lymph node lymphomyomalosis is challenging to detect. It is occult. It is occasionally found in lymph nodes of gynecological tumors and surgical stages of urinary system malignant tumors, including (13–15) of the uterus, oviduct, ovarian cancer, or bladder cancer. The total incidence rate in gynecologic malignant tumor staging surgery is 1.3% (16). It has been found that most extrapulmonary LAM occurs in lymph nodes along the mediastinum or retroperitoneal lymphatics, with three main locations followed by the posterior mediastinum, the upper retroperitoneal region close to the abdominal aorta, and the pelvis (17). These three places are all candidate sites for primary LAM, but there is some evidence that LAM originates in the pelvis and moves along the lymphatic direction (18).

The origin of LAM is still under study. Hayashi et al. reported that 90% of patients with pulmonary LAM have uterine LAM, and all uterine LAM lesions are accompanied by LAM lesions of retroperitoneal or pelvic lymph nodes. Therefore, it is assumed that the uterus or the area near the retroperitoneal or pelvic cavity may be the primary site for LAM, and LAM cells can spread to the lung through lymphatic vessels (4). At the same time, Suzuki et al. found that LAM originated in uterine smooth muscle and then invaded retroperitoneal lymph nodes through lymphatic circulation (19), which is more consistent with what Hayashi et al. said. However, since LAM also occurs in men (20), there should be other potential sources.

In this case, only pelvic lymph node LAM was found, and no lesions were found in the uterus or lungs. Currently, pelvic lymph nodes are the primary site. Whether they can develop into pulmonary LAM remains to be further followed up. The relationship between lung LAM and lymph node LAM is not clear. Some scholars found that in patients without symptoms and signs of lung LAM, lymph node LAM may be a high-risk indicator for the development of lung LAM (17). Some scholars also believe that occult lymph node LAM found in the surgical staging of gynecological cancer has nothing to do with lung LAM and TSC. However, these two diseases still need to be evaluated in detail (13).

We summarized some cases of sporadic lymph node LAM during surgery. We found that only a tiny portion (2/61) developed pulmonary LAM, the vast majority did not develop pulmonary LAM, and the association with TSC was very low (2/61) (see Table 1) (13–15, 18–22). As reported in this case, we were followed up for 12 months, but there was no significant pulmonary LAM. However, due to the slow progression of the disease, there is also the possibility that the follow-up time is not long enough, and lung involvement does not appear. According to Matsui et al., it may take 1–2 years for lymph node LAM to develop into pulmonary LAM (17), and the size of lymph node lesions in patients with pulmonary LAM is at least 10 mm. Kuno et al. (21) reported that a woman with <10 mm of lymphadenopathy developed pulmonary LAM 7 years after the operation. Therefore, lymph node LAM may be a high-risk factor for the development of pulmonary LAM. However, for more than 1–2 years without developing pulmonary LAM, it may take longer and may be related to the size of lymph node lesions.

**Table 1:** Female cases of pelvic and peritoneal lymph node LAM found during the operation.

**Case report**	**Number of patients involved**	**Age, median and range**	**Disease type**	**Immunohisto chemistry**	**TSC**	**Develop into lung LAM**	**Follow up time, median and range**	**Prognosis**
Iwasa et al. (15)	*three cases*	*59 years old, (47-71 years old)*	*one case of cervical squamous cell carcinoma and two cases of endometrial adenocarcinoma*	*α-SMA, desmin, HMB45, ER, PR, D2-40 were positive*	*Nothing*	*No*	*27.3 months, (20*–*39 months)*	*All survived*
School meester et al. (14)	*19 cases*	*56.3 years old (35-71 years old)*	*five cases of ovarian cancer, nine cases of uterine body cancer, one case of adult ovarian granulosa cell tumor, one case of cervical squamous cell carcinoma, one case of high-grade serous carcinoma of the fallopian tube, one case of hysteromyoma and one case of sporadic LAM*	*HMB45, β-catenin were positive*	*Nothing*	*No*	*33.8 months, (3*–*123 months)*	*One case died of uterine serous carcinoma 23 months after the operation, one case of sporadic LAM persisted, and the rest survived*.
Kuno et al. (21)	*eight cases*	*56 years old (36-74 years old)*	*four cases of uterine body cancer, two cases of ovarian cancer and two cases of cervical cancer*	*α-SMA, HMB45 were positive*	*Nothing*	*one case*	*26 months, (4*–*167 months)*	*All survived*
Ando et al. (18)	*three cases*	*46.7 years old, (44-49 years old)*	*one case of endometrial carcinoma and two cases of hysteromyoma*	*α-SMA, HMB45, D2-40 was positive*	*Nothing*	*No*	*11.3 months (4*–*18 months)*	*All survived*
Remo et al. (22)	*one case*	*44 years old*	*Cervical mucinous adenocarcinoma*	*α-SMA,HMB45 were positive*	*Nothing*	*Yes*	*10 months*	*survived*
Suzuki et al. (19)	*one case*	*47 years old*	*Endometrial carcinoma is complicated by systemic lupus erythematosus*.	*α-SMA, caldesmon, Melan A, HMB45 and ER were positive*	*Nothing*	*No*	*Not specified*	*survived*
Rabban et al. (13)	*26 cases*	*56 years old (31-79 years old)*	*17 cases of uterine cancer, five cases of ovarian cancer, three cases of cervical cancer, one case of bladder cancer*	*α-SMA(23/23), HMB45(24/25), ER and D2-40 were positive*	*2 cases*	*No*	*18 months (3*–*156 months)*	*All survived; one case had a local recurrence of uterine body cancer*.

In addition, LAM mainly occurs in women of childbearing age and easily worsens during pregnancy. It is known that LAM cells express estrogen and progesterone receptors simultaneously, and a study found that the expression of PR is usually higher than that of ER (23), so female hormones play an essential role in the development and progression of the disease. However, many postmenopausal women also have LAM, and the course of disease of postmenopausal women with LAM is relatively stable (24). The patient, in this case, has been postmenopausal for 1 year. It is worth mentioning that the patient was confirmed to have high-grade serous carcinoma of the fallopian tube by postoperative pathology. Primary fallopian tube carcinoma (PFTC) is a rare malignant tumor in women, accounting for 0.14–1.8% of all female reproductive tract malignant tumors (25). Her ovaries are in a non-functional state, and we found that ER and PR were negative when staining pathological specimens (see Figures 2E,F). Therefore, hormone dependence may not be applicable in this case. It is speculated that the patient may have had pelvic lymph node LAM before menopause, but it has not been found. However, the condition is relatively stable after menopause, and there is no clinical manifestation of LAM. It is also possible that the patient developed pelvic lymph node LAM after menopause. However, pelvic and peritoneal lymph node LAM is also found in postmenopausal endometrial cancer (13–15). Most patients with endometrial cancer are in a state of relative excess estrogen, indicating that hormone levels still play an essential role in the occurrence and development of the disease.

Although LAM is hormone-dependent, it is interesting that LAM is not unique to women and, in rare cases, exists in men (20). Just as endometriosis is also a hormone-dependent disease common in women, it is now found to be not unique to women and sporadic in men (26). Combined with the metastatic characteristics of the disease, we believe that the disease has certain similarities with endometriosis in occurrence and development. They are hormone-dependent and have malignant biological behaviors such as migration and invasion. At the same time, most of the common and incidental parts of the two are similar, but the treatment methods of the two are different. Therefore, can the pathogenesis of endometriosis be used to explain the occurrence of the disease? As far as this case is concerned, this idea is difficult to realize and is not mature. We just put forward a new research idea, and more and broader clinical data are still needed for summary and analysis.

## Conclusion

We report a case of sporadic pelvic lymph node LAM during staged surgery for primary fallopian tube cancer. At present, the source and pathogenesis of LAM cells are not clear. We propose that the disease is similar to endometriosis in hormone dependence and biological behavior. At the same time, the relationship between sporadic lymph node LAM and pulmonary LAM remains uncertain. It may be only a matter of time for sporadic lymph node LAM to develop into intrapulmonary LAM. It is also possible that the two are not related. At this stage, the long-term follow-up of internal medicine and gynecologists is essential for the correct diagnosis of LAM and cancer-related diseases.

## Data Availability Statement

The original contributions presented in the study are included in the article/supplementary material, further inquiries can be directed to the corresponding author/s.

## Ethics Statement

The study was approved by the Institutional Review Board of the LongHua Hospital Affiliated to Shanghai University of Traditional Chinese Medicine. Written informed consent was obtained from the patient for the publication of this case report and for the publication of any potentially identifiable data or images.

## Author Contributions

SX and YC have made great contributions to the collation and analysis of case data, conception and design, and writing manuscripts. QT and LX participated in the clinical diagnosis, treatment, and follow-up of patients. LZ and ZW participated in the literature review and manuscript drafting. EY is the main surgery, which has made great contributions to the formulation of a patient's clinical diagnosis and treatment plan and the revision of manuscripts. All authors contributed to the article and approved the submitted version.

## Funding

This research was supported by the Joint Research Project on Emerging Frontier Technologies for Shanghai Municipal Hospitals (No. SHDC12019106), the National Natural Science Foundation of China Projects (No. 81704105), the Fourth Batch of Seedling Raising Plan Projects of LongHua Hospital Affiliated to Shanghai University of Traditional Chinese Medicine (No. YM2021006).

## Conflict of Interest

The authors declare that the research was conducted in the absence of any commercial or financial relationships that could be construed as a potential conflict of interest.

## Publisher's Note

All claims expressed in this article are solely those of the authors and do not necessarily represent those of their affiliated organizations, or those of the publisher, the editors and the reviewers. Any product that may be evaluated in this article, or claim that may be made by its manufacturer, is not guaranteed or endorsed by the publisher.

## References

[1] McCarthy C Gupta N Johnson SR Yu JJ McCormack FX. Lymphangioleiomyomatosis: pathogenesis, clinical features, diagnosis, and management. Lancet Respir Med. (2021) 9:1313–27. 10.1016/S2213-2600(21)00228-9 34461049

[2] McCormack FX Gupta N Finlay GR Young LR Taveira-DaSilva AM Glasgow CG. Official American Thoracic Society/Japanese Respiratory Society Clinical Practice Guidelines: lymphangioleiomyomatosis diagnosis and management. Am J Respir Crit Care Med. (2016) 194:748–61. 10.1164/rccm.201607-1384ST 27628078 PMC5803656

[3] Harknett EC Chang WY Byrnes S Johnson J Lazor R Cohen MM. Use variability in national and regional data to estimate the prevalence of lymphangioleiomyomatosis. QJM. (2011) 104:971–9. 10.1093/qjmed/hcr116 21764810

[4] Hayashi T Kumasaka T Mitani K Terao Y Watanabe M Oide T. Prevalence of uterine and adnexal involvement in pulmonary lymphangioleiomyomatosis: a clinicopathologic study of 10 patients. Am J Surg Pathol. (2011) 35:1776–85. 10.1097/PAS.0b013e318235edbd 22020043

[5] Ryu JH Moss J Beck GJ Lee JC Brown KK Chapman JT. The NHLBI lymphangioleiomyomatosis registry: characteristics of 230 patients at enrollment. Am J Respir Crit Care Med. (2006) 173:105–11. 10.1164/rccm.200409-1298OC 16210669 PMC2662978

[6] Taveira-DaSilva AM Jones AM Julien-Williams P Yao J Stylianou M Moss J. Severity and outcome of cystic lung disease in women with tuberous sclerosis complex. Eur Respir J. (2015) 45:171–80. 10.1183/09031936.00088314 25537563 PMC8356806

[7] Yeoh ZW Navaratnam V Bhatt R McCafferty I Hubbard RB Johnson SR. Natural history of angiomyolipoma in lymphangioleiomyomatosis: implications for screening and surveillance. Orphanet J Rare Dis. (2014) 9:151. 10.1186/s13023-014-0151-3 25277108 PMC4203902

[8] Zhe X Schuger L. Combined smooth muscle and melanocytic differentiation in lymphangioleiomyomatosis. J Histochem Cytochem. (2004) 52:1537–42. 10.1369/jhc.4A6438.2004 15557209

[9] Krymskaya VP. Smooth muscle-like cells in pulmonary lymphangioleiomyomatosis. Proc Am Thorac Soc. (2008) 5:119–26. 10.1513/pats.200705-061VS 18094094 PMC2645298

[10] Bittmann I Rolf B Amann G Löhrs U. Recurrence of lymphangioleiomyomatosis after single lung transplantation: new insights into pathogenesis. Hum Pathol. (2003) 34:95–8. 10.1053/hupa.2003.50 12605373

[11] Szpurek D Szubert S Zielinski P Frankowski A Sajdak S Moszynski R. Malignant presentation of uterine lymphangioleiomyomatosis. Taiwan J Obstet Gynecol. (2015) 54:603–7. 10.1016/j.tjog.2015.08.015 26522119

[12] Krymskaya VP McCormack FX. Lymphangioleiomyomatosis: A Monogenic Model of Malignancy. Annu Rev Med. (2017) 68:69–83. 10.1146/annurev-med-050715-104245 28099079 PMC5663315

[13] Rabban JT Firetag B Sangoi AR Post MD Zaloudek CJ. Incidental pelvic and para-aortic lymph node lymphangioleiomyomatosis detected during surgical staging of pelvic cancer in women without symptomatic pulmonary lymphangioleiomyomatosis or tuberous sclerosis complex. Am J Surg Pathol. (2015) 39:1015–25. 10.1097/PAS.0000000000000416 25786086

[14] Schoolmeester JK Park KJ. Incidental nodal lymphangioleiomyomatosis is not a harbinger of pulmonary lymphangioleiomyomatosis: a study of 19 cases with evaluation of diagnostic immunohistochemistry. Am J Surg Pathol. (2015) 39:1404–10. 10.1097/PAS.0000000000000470 26135558 PMC4976695

[15] Iwasa Y Tachibana M Ito H Iwami S Yagi H Yamada S. Extrapulmonary lymphangioleiomyomatosis in pelvic and paraaortic lymph nodes associated with uterine cancer: a report of 3 cases. Int J Gynecol Pathol. (2011) 30:470–5. 10.1097/PGP.0b013e318212e1e6 21804397

[16] Nagasaka T Murakami Y Sasaki E Hosoda W Nakanishi T Yatabe Y. Minute perivascular epithelioid cell (PEC) nests in the abdominal lymph nodes–a putative precursor of PEComa. Pathol Int. (2015) 65:193–6. 10.1111/pin.12262 25677636

[17] Matsui K Tatsuguchi A Valencia J Yu ZX Bechtle J Beasley MB. Extrapulmonary lymphangioleiomyomatosis (LAM): clinicopathologic features in 22 cases. Hum Pathol. (2000) 31:1242–8. 10.1053/hupa.2000.18500 11070117

[18] Ando H Ogawa M Watanabe Y Tsurunaga K Nakamura C Tamura H. Lymphangioleiomyoma of the uterus and pelvic lymph nodes: a report of 3 cases, including the potentially earliest manifestation of extrapulmonary lymphangioleiomyomatosis. int J Gynecol Pathol. (2020) 39:227–32. 10.1097/PGP.0000000000000589 30789500

[19] Suzuki K Nagasaka K Oda K Abe H Maeda D Matsumoto Y. A case of lymphangioleiomyomatosis associated with endometrial cancer and severe systemic lupus erythematosus. BMC Cancer. (2016) 16:390. 10.1186/s12885-016-2413-z 27377753 PMC4932736

[20] Schiavina M Di Scioscio V Contini P Cavazza A Fabiani A Barberis M. Pulmonary lymphangioleiomyomatosis in a karyotypically normal man without tuberous sclerosis complex. Am J Respir Crit Care Med. (2007) 176:96–8. 10.1164/rccm.200610-1408CR 17431222

[21] Kuno I Yoshida H Shimizu H Uehara T Uno M Ishikawa M. Incidental lymphangioleiomyomatosis in the lymph nodes of gynecologic surgical specimens. Eur J Obstet Gynecol Reprod Biol. (2018) 231:93–7. 10.1016/j.ejogrb.2018.10.027 30336310

[22] Remo A Zanella C Parcesepe P. Diagnostic management of occult nodal lymphangioleiomyomatosis detected during pelvic cancer staging. Localized finding or systemic disease? Sarcoidosis Vasc Diffuse Lung Dis. (2019) 36:33–8. 32476934 10.36141/svdld.v36i1.7110 PMC7247108

[23] Gao L Yue MM Davis J Hyjek E Schuger L. In pulmonary lymphangioleiomyomatosis expression of progesterone receptor is frequently higher than that of estrogen receptor. Virchows Arch. (2014) 464:495–503. 10.1007/s00428-014-1559-9 24570392

[24] Johnson SR Tattersfield AE. Decline in lung function in lymphangioleiomyomatosis: relation to menopause and progesterone treatment. Am J Respir Crit Care Med. (1999) 160:628–33. 10.1164/ajrccm.160.2.9901027 10430739

[25] Pectasides D Pectasides E Economopoulos T. Fallopian tube carcinoma: a review. Oncologist. (2006) 11:902–12. 10.1634/theoncologist.11-8-902 16951394

[26] Al-Obaidy KI Idrees MT. endometriosis with cystic degeneration: a rare disease of males. Int J Surg Pathol. (2019) 27:311–4. 10.1177/1066896918797438 30178697

